# Modified Nuss operation using introducer-bar complex for pectus excavatum in adults: a retrospective study

**DOI:** 10.1186/s13019-021-01624-6

**Published:** 2021-09-22

**Authors:** Lei Wang, Juan Liu, Yao Li, Tienan Feng, Beibei Cao, Haibo Xiao, Fengqing Hu, Guoqing Li

**Affiliations:** 1 Department of Cardiothoracic Surgery, Xinhua Hospital Affiliated To Shanghai Jiao Tong University School of Medicine, Shanghai, 200092 China; 2 Department of Cardiovascular Medicine, Baoshan Branch of Shanghai Shuguang Hospital, Shanghai University of Traditional Chinese Medicine, Shanghai, 201900 China; 3 Department of Disaster and Emergency Medicine, Shanghai East Hospital, Tongji University, 1239 Siping Road, Shanghai, 200120 China; 4 Clinical Research Institute, Shanghai Jiao Tong University School of Medicine, Shanghai, 200025 China; 5 Second Affiliated Hospital of Chengdu Medical College, China National Nuclear Corporation, 416 hospital, Chengdu, 610051 Sichuan China; 6 Department of Printing Equipment Engineering, Shanghai Publishing and Printing College, Shanghai, 200093 China

**Keywords:** Adult Nuss operation, Introducer-bar complex, Pectus excavatum, Surgical correction

## Abstract

**Background:**

Shortcoming of traditional Nuss operation on adults is gradually found in the clinical practice. A new kind of introducer-bar complex was introduced. However, there is limited evidence regarding its safety and efficacy. Therefore, a single center, retrospective study was conducted to address this issue.

**Methods:**

Patients with pectus excavatum who underwent surgery between January 2015 and June 2017 were consecutively enrolled in this study. In all, 52 patients underwent the modified procedure using the introducer-bar complex (new procedure group), whereas 48 underwent the traditional anti-Nuss procedure (traditional procedure group). Outcomes analysis of balanced baseline was performed to compare the intraoperative and postoperative short-term outcomes.

**Results:**

All patients in the new procedure group had shorter operation duration (51.54 ± 20.32 vs. 79.45 ± 13.88 min, *p* = 0.017), postoperative hospitalizations (4.77 ± 1.62 vs. 6.86 ± 2.18 days, *p* = 0.028), plate removal surgery durations (39.30 ± 8.97 vs. 60.30 ± 10.49 min, *p* < 0.001), and less blood loss during operation (6.25 ± 4.88 vs. 10.90 ± 5.75 ml, *p* = 0.003) than patients in the traditional procedure group. There was no significant difference in the length of incision, postoperative Haller index, cost, number of steel bars, postoperative surgical outcome and incidence of complications between the two groups.

**Conclusion:**

Through the main clinical outcome were similar, our results shown that modified procedure may have the shorter operation time, postoperative hospital stay, and operation time for plate removal and less blood loss, which may bring potential clinical benefits to patients.

**Supplementary Information:**

The online version contains supplementary material available at 10.1186/s13019-021-01624-6.

## Introduction

Pectus excavatum (PE) is the most common congenital deformity of the anterior wall of the chest, affecting 1–8 per 1000 live births. The minimally invasive repair of pectus excavatum without any cartilage resection was primarily developed for children, but it has gained more and more widespread acceptance in adults [1]. First attempt to correct PE was performed in 1911 by Ludwig Meyer and Ravitch procedure was subsequent reported since 1949 [2, 3]. After that, Nuss procedure (NP) and several technical modifications by the placement of metal bars to lift the depressed chest wall was introduced and considered as popular minimally invasive technique to repair PE [2, 4, 5]. However, some disadvantages with the Nuss procedure was gradually found. Firstly, the steel plate without curved part was required to be shaped with special tools before the operation leading to the increase of operation time. Secondly, it was difficult and traumatic when turning over the steel bar in the traditional Nuss procedure. Finally, it was hard to fix the steel plate firmly just by steel wire and it took a long period for surgeons to place or extract of the steel plate [6, 7].

To address the above shortcomings, we designed a new kind of introducer-bar complex [8]. The new kind of steel bar was classified as 15 different specification and could not be shaped. One end of the steel bar was designed to connect with the introducer, so the introducer-bar complex was made. The introducer-bar complex could be installed or removed by being pushed in or pulled out through the tunnel without being turned over widely, which was easier and less invasive than in traditional Nuss procedure. Moreover, the bar could be fixed more firmly by screws as well as steel wires and fixing pieces.

The aim of our study was to explore the safety and efficacy of modified Nuss operation using introducer-bar complex for adult patients with PE using a single center, retrospective study.

## Patients and methods

### Patient groups

This is a single center clinical cohort study based on retrospective analysis of prospectively collected data on patients with pectus excavatum who underwent surgical correction from January 2015 to June 2017, total 100 patients were identified from databases. 52 cases received modified Nuss procedure using introducer-bar complex as the new procedure group, while 48 cases received traditional Nuss procedure as the traditional procedure group. The type of operation selected by the patient depended on the will of the family and the indication of the operation. Preoperative examination of patients included blood routine, electrolyte, liver and kidney function, heart color ultrasound, chest CT, electrocardiogram and pulmonary function.

The patients included in this study were at least 18 years old. Exclusion criteria were severe lung disease, pre-existing heart disease and those both with pectus excavatum and pectus carinatum. The patient was followed up within 3 months after operation to observe the wound healing and the displacement of the steel plate by the chest CT. Then, the chest CT was checked every 6 months until the remove of the steel plate. The study protocol was approved by the Institutional Review Board of Xinhua Hospital (Approval No. XHEC-D-2020-062), Shanghai Jiao Tong University School of Medicine, and informed consent was received from all patients.

### New kind of introducer-bar complex and accessories

**Fig. 1:**
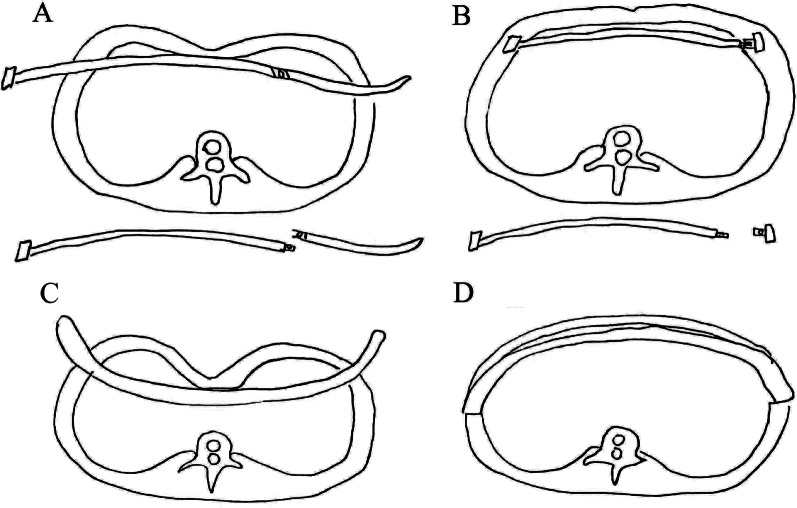
Introducer-bar complex configuration and accessories. Diagram of the new modified Nuss procedure (**a**, **b**) and the traditional Nuss procedure (**c**, **d**)

Unlike traditional Nuss bar, the new kind of steel bar was curved according to the normal structure of the human anterior chest wall and included 15 different specifications distinguished by the different lengths that varied from 12 to 26 cm. One end of the steel bar was fused with a bar stabilizer, and the other end was designed to connect with the introducer or stabilizer. Once the bar was connected to the introducer, introducer-bar complex was ready (Fig. 1). Therefore, obvious difference could be found between the new kind and traditional steel bar. For one thing, we do not need to bend the new kind steel bar during the operation because the bar has been produced preoperatively and classified as 15 different specifications. So that the procedure is simplified and the damage to the steel bar is avoided. For another, we do not have to turn over the new kind of introducer-bar complex widely but push it in or pull it out through the tunnel as the steel bar and stabilizer could be connected directly and fixed by screws. As a result, soft tissue injury is decreased and the operation time is reduced. Finely, middle part of the new steel bar was changed as rough surface so as to effectively increase the friction between the bar and the contact tissue, and then we can fix the steel bar more firmly.

### Modified surgical procedure

All patients were anesthetized with general intravenous anesthesia and underwent tracheal intubation. The steel bar can be selected from the bar box (Additional file 2: Figure S1). Before the operation, we measure the distance A between the left anterior axillary lines of the intercostal level to the lowest point of the sternum. Then distance B between the right anterior axillary lines of the intercostal level to the lowest point of the sternum was also measured. Size of the bar may be caculated as distance A plus distance B plus 2–3 cm roughly. The bar can be changed during the operation if the size was not appropriate. Bilateral vertical skin incisions were made between the anterior axillary and medial axillary lines, in line with the deepest point of the depression. A 5-mm-diameter thoracoscope was inserted into the right thoracic cavity through the right incision to guide and monitor the procedure. Tied the steel bar to introducer so the introducer-bar complex was made. The introducer-bar complex was inserted into the right thoracic cavity and woven behind the sternum anterior to the pericardium through the bilateral pleural cavity. Then, the introducer-bar complex was pushed in through the tunnel without turning it over widely, and the deformity was corrected. Separate the introducer-bar complex and remove the introducer. A stabilizer was placed on the left side of the bar and was used to fix the bar. Finally, bilateral stabilizers were tied to the ribs and intercostal muscles with wire to avoid bar rotation (see the Additional file 1: Video). For the traditional procedure group, traditional Nuss operation was performed.

### Statistical analysis

Epidata 3.1 was used for data entry; and SPSS 20.0 statistical software were used for data analysis. Basic information included sex, age, type of operation, pectus excavatum (PE) and preoperative Haller index. We evaluated the outcomes as follows: sternum depression evaluated by Chest CT scan, the symmetry of the chest wall morphology, depression and the satisfactions of patient and their families: the thorax appears full with good extension and elasticity. The outcomes were considered excellent, good, fair, or poor if 4, 3, 2, or 1/0 criteria were positive, respectively. The continuous variables with normal distribution are expressed by mean ± standard deviation. The continuous variables with non-normal distribution are expressed by medians and inter-quartile ranges (IQRs). The count data are expressed as number of cases (n) and percentage (%). The comparisons among normally distributed continuous variables were conducted via t-test or ANOVA, whereas those among non-normally distributed variables were conducted via Mann–Whitney U test or Kruskal–Wallis test. Comparisons between enumeration data were conducted by Chi-Square or Fisher exact method. Finally, *P* < 0.05 was considered to be statistically significant.

## Results

### Study characteristics

All of the 100 cases were successfully treated. In the new procedure group, there were 41 males and 11 females with an average age of 22.51 ± 5.12 ranging from 18 to 42 years old. The type of symmetry (n = 36) accounted for 69.23% of all patients (n = 52), and the preoperative Haller index was 4.08 ± 0.90. In the traditional procedure group, there were 38 males and 10 females with an average age of 21.52 ± 4.61 ranging from 18 to 40 years old. The type of symmetry (n = 32) accounted for 66.67% of all patients (n = 43), and the preoperative Haller index was 4.12 ± 0.88. There were no significant difference in age, sex, type and the preoperative Haller index between the two groups (*p* > 0.05) (Table 1).

**Table 1:** Comparison of patient information between new procedure and traditional procedure group

	New procedure group (n = 52)	Traditional procedure group (n = 48)	*p* value
Sex			0.969
Male	41 (78.85%)	38 (79.17%)	
Female	11 (21.15%)	10 (20.83%)	
Age (years)	22.51 ± 5.12	21.52 ± 4.61	0.356
Type			0.784
Symmetry	36(69.23%)	32(66.67%)	
Asymmetry	16(30.77%)	16(33.33%)	
Pectus excavatum **(**PE)			1.000
Primarily PE	48 (92.31%)	45 (93.75%)	
Recurrent PE	4 (7.69%)	3 (6.25%)	
Preoperative Haller index	4.08 ± 0.90	4.12 ± 0.88	0.844

### Intraoperative and short-term outcome

**Fig. 2:**
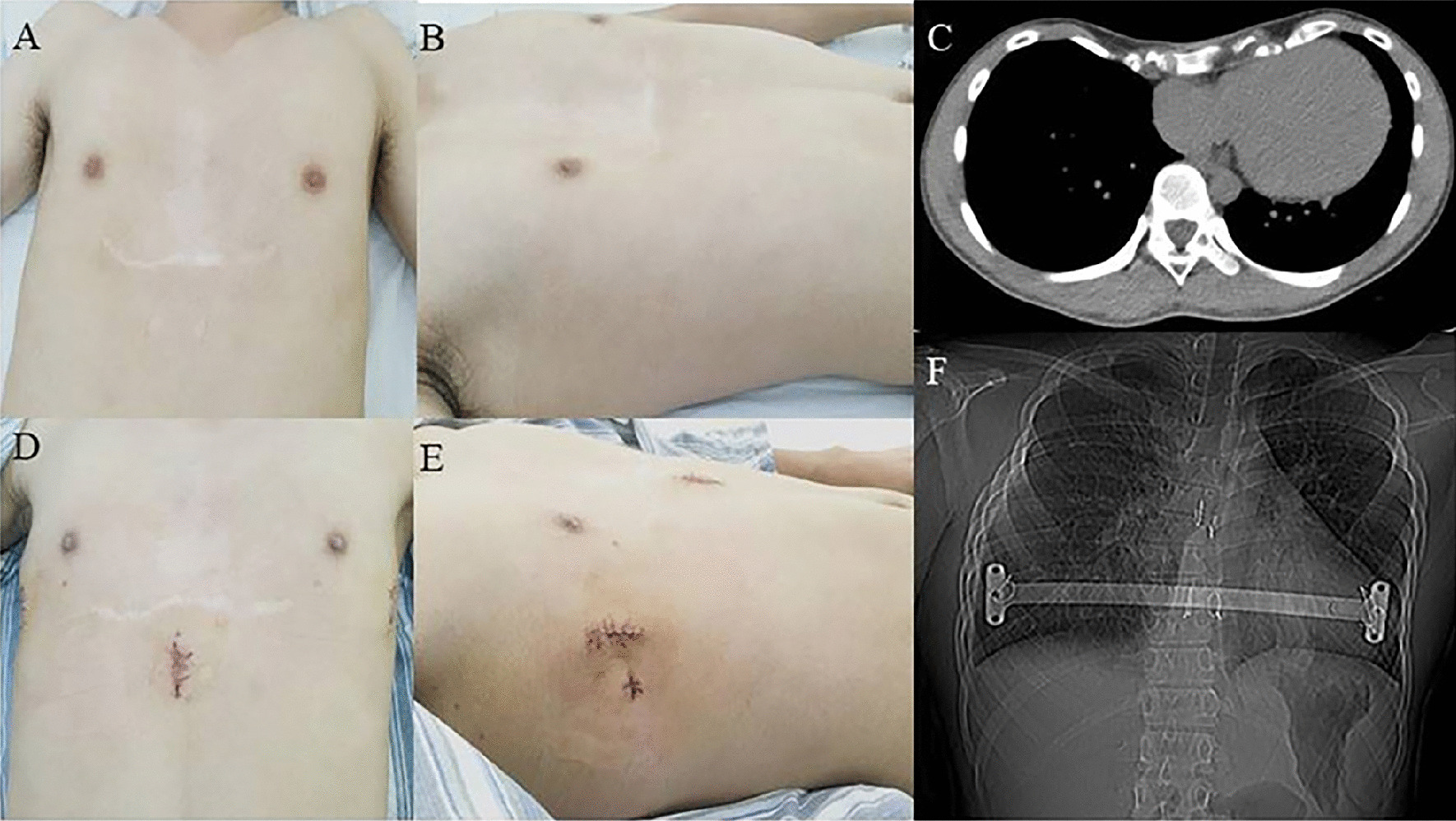
Appearance and chest scan of a 19-year-old recurrent PE patient before and after modified Nuss procedure with introducer-bar complex

**Fig. 3:**
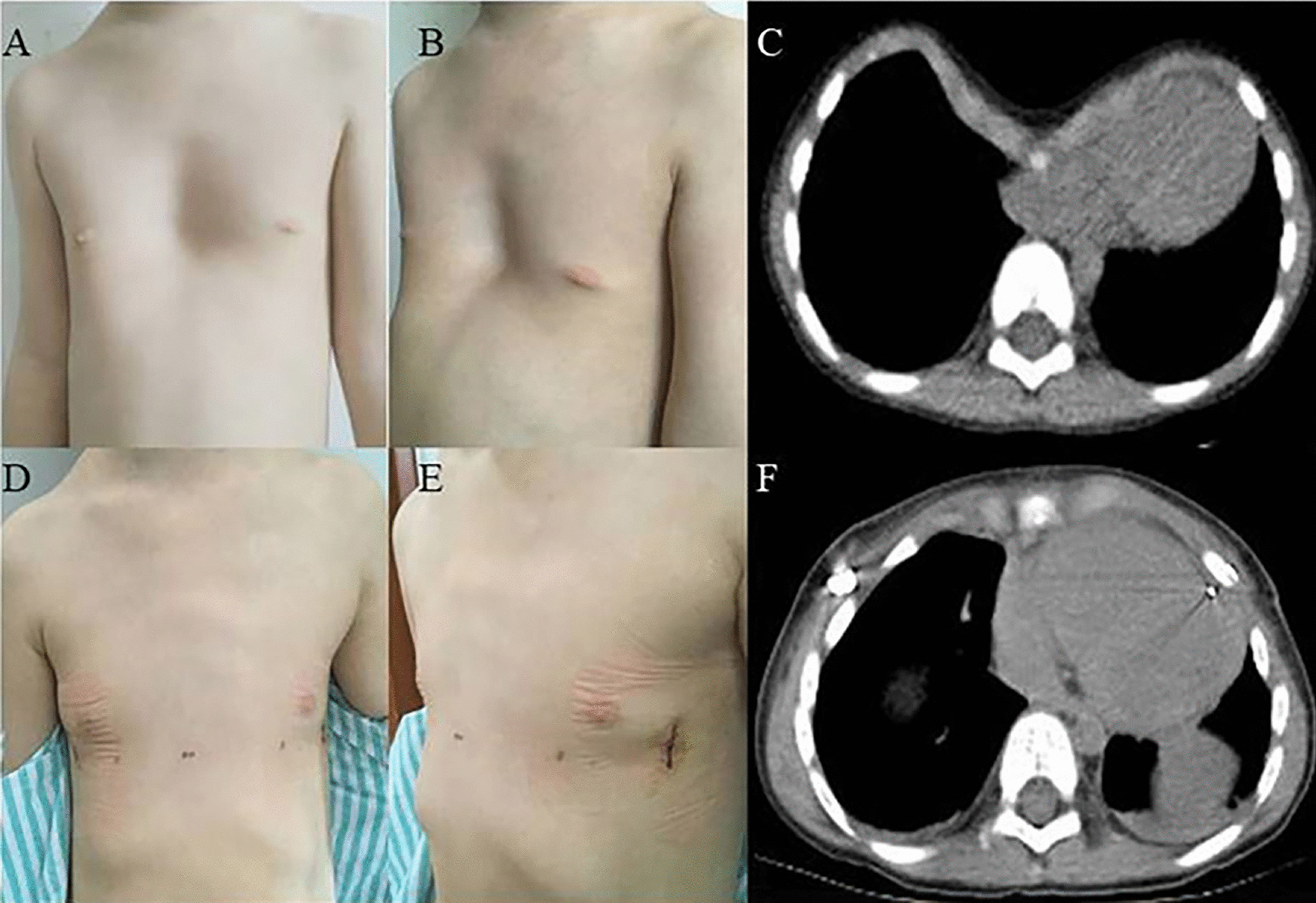
Appearance and chest scan of a 18-year-old PE patient before and after modified Nuss procedure with introducer-bar complex

**Fig. 4:**
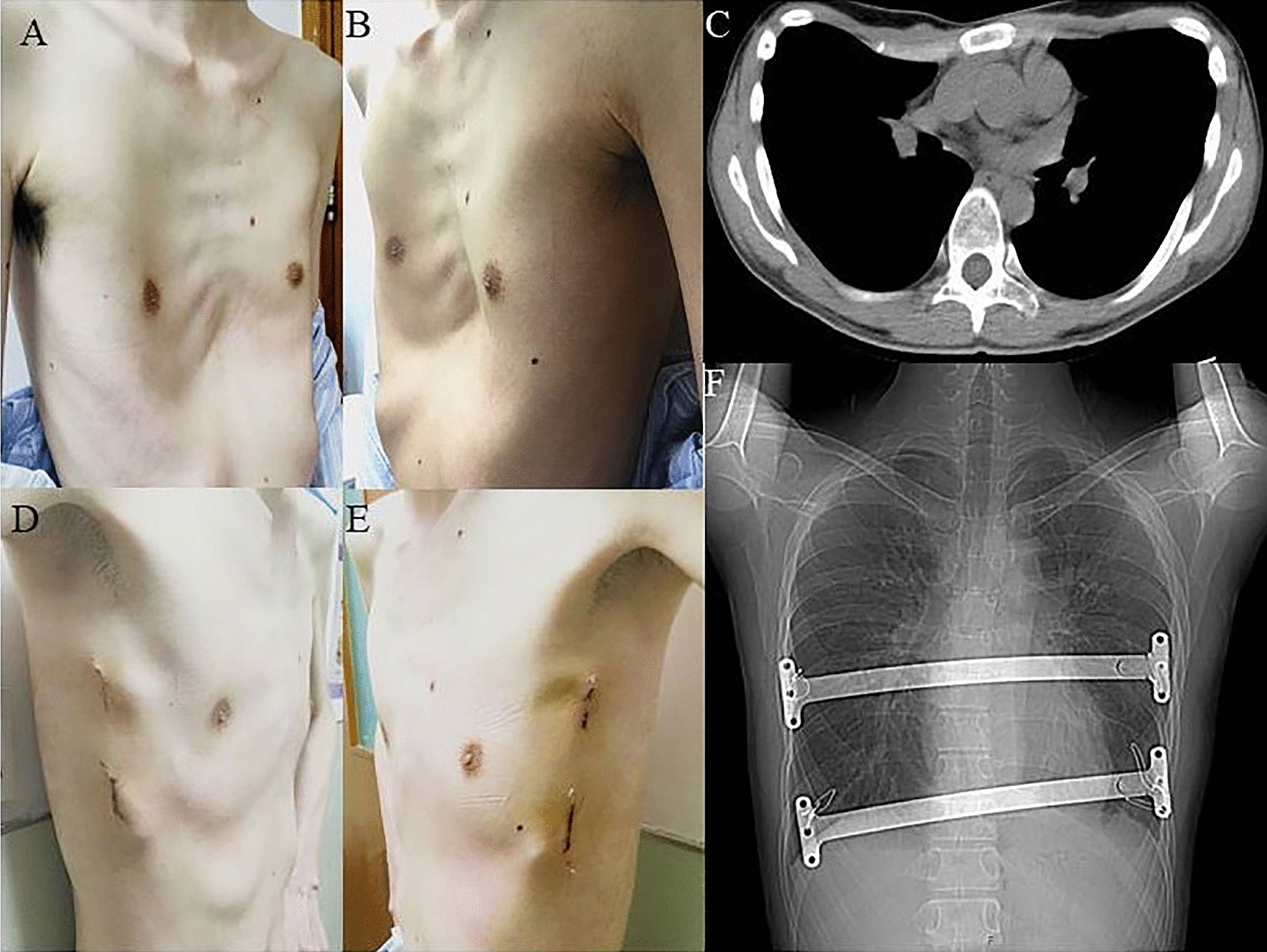
Appearance and chest scan of a 31-year-old PE patient with 2 bars before and after modified Nuss procedure with introducer-bar complex

In the new procedure group, the operation time was 51.54 ± 20.32 min, the length of incision was 3.35 ± 0.38 cm, the blood loss was 6.25 ± 4.88 ml, the postoperative Haller index was 2.86 ± 0.28, the postoperative hospital stay was 4.77 ± 1.62 days, the cost was 57,200.00 ± 1125.00 yuan, and the operation time for plate removal was 39.30 ± 8.97 min. There were 48 patients with primarily PE and 4 patients with recurrent PE (Fig. 2). 45 PE patients were treated by 1 bar (Fig. 3) (Additional file 3: Figure S2), and 7 PE patients were treated by 2 bars (Fig. 4). The postoperative surgical outcome was good in 43 patients (82.69%) and fair in 9 patients (17.31%).

In the traditional procedure group, the operation time was 79.45 ± 13.88 min, the length of incision was 3.42 ± 0.44 cm, the blood loss was 10.90 ± 5.75 ml, the postoperative Haller index was 2.91 ± 0.32, the postoperative hospital stay was 6.86 ± 2.18 days, the cost was 56,383.00 ± 1045.00yuan, and the operation time for plate removal was 60.30 ± 10.49 min. There were 45 patients with primarily PE and 3 patients with recurrent PE.42 PE patients were treated by 1 bar, and 6 PE patients were treated by 2 bars. The postoperative surgical outcome were good in 39 patients (81.25%) and fair in 9 patients (18.75%).

There were no significant difference in the length of incision, the postoperative Haller index, the cost, number of steel bars, and the postoperative surgical outcome between the two groups (*p* > 0.05) (Table 2). However, less blood loss during the operation as well as shorter operation time, postoperative hospital stay and operation time for plate removal of the modified procedure were found with acceptable postoperative surgical results (Table 2) (*p* < 0.05).

**Table 2:** Comparison of surgical characteristics between new procedure and traditional procedure group

	New procedure group (n = 52)	Traditional procedure group (n = 48)	P value
Operation time (min)	51.54 ± 20.32	79.45 ± 13.88	0.017
Length of incision (cm)	3.35 ± 0.38	3.42 ± 0.44	0.767
Blood loss	6.25 ± 4.88	10.90 ± 5.75	0.003
Postoperative Haller index	2.86 ± 0.28	2.91 ± 0.32	0.906
Postoperative hospital stay (day)	4.77 ± 1.62	6.86 ± 2.18	0.028
Cost (yuan)	57,200.00 ± 1125.00	56,383.00 ± 1045.00	0.838
Number of steel bars			0.800
1	45 (86.54%)	42(87.50%)	
2	7 (13.46%)	6 (12.50%)	
Operation time for plate removal (min)	39.30 ± 8.97	60.30 ± 10.49	0.000
Postoperative surgical outcome			0.716
Good	43 (82.69%)	39 (81.25%)	
Fair	9 (17.31%)	9 (18.75%)	
Poor	0 (0.00%)	0 (0.00%)	

## Complications

There was no significant difference in the incidence of complications between the two groups (*p* > 0.05). In the new procedure group, the complications after operation included 1 cases of wound infection, 1 case of pneumothorax which needed drainage, 1 case with bar displacement and 1 case with bar exposure due to delayed wound healing. There was also 1 patient with atelectasis who need aspiration of sputum through bronchoscopy. Debridement was performed for the 2 cases with wound infection and the case with bar exposure due to delayed wound healing. The patient with obvious bar displacement required reoperation and repositioning of the bar outside the theproximal rib with wire. The rest of the patients recover smoothly.

In the traditional procedure group, the complications after operation included 1 cases of pneumothorax need to be treated, 1 cases of pleural effusion which needed drainage, 2 cases of wound infection or bar exposure due to delayed wound healing (all of the 2 received debridement), and 1 case with bar displacement (reoperation and repositioning of the bar was also performed). There was also 2 patient with atelectasis who need aspiration of sputum through bronchoscopy. There was no significant difference in complications (*p* > 0.05) (Table 3).

**Table 3:** Complications in new procedure and traditional procedure group

	New procedure group **(**n = 52)	Traditional procedure group **(**n = 48)	*p* value
Complications			0.445
No	47 (90.38%)	41 (85.42%)	
Yes	5 (9.62%)	7 (14.58%)	
Pneumothorax which needed drainage	1 (20.00%)	1 (20.00%)	
Pleural effusion which needed drainage	0 (0.00%)	1 (0.00%)	
Atelectasis which need aspiration of sputum through bronchoscopy	1 (20.00%)	2 (20.00%)	
Wound infection	1 (20.00%)	1 (40.00%)	
Bar displacement	1 (20.00%)	1 (20.00%)	
Bar exposure due to delayed wound healing	1 (20.00%)	1 (20.00%)	

## Discussion

In the past 10 years, Nuss bar and traditional Nuss procedure have been used for correction of PE [9–11]. However, shortcoming of the bar and procedure was also found [12, 13]. For example, the steel bar needs to be shaped with special tools before the operation and it is usually hard to push the steel bar through the anterior sternum [14]. Besides, it is often difficult for surgeons to fix the steel bar firmly just by steel wire and it usually took a long time to place or withdraw the bar [15]. As a result, we invent the introducer-bar complex and modify the traditional procedure to achieve better result.

Compared with traditional Nuss bar, this novel introducer-bar complex has several advantages. Firstly, the new steel bar was produced before the operation and was connected to the introducer. Therefore, there is no need to bend the steel bar during the operation which may damage the bar and lead to potential recurrence of pectus excavatum. Secondly, the introducer-bar complex is installed or removed by pushing or pulling without flipping widely, which simplify the procedure and decreases intraoperative trauma. Thirdly, traditional steel bar was fixed only by steel wire in the previous procedure, however, new steel bar could also be fixed by screws because of the arrangement of screw holes which can be used to fix the steel plate with the help of screws and a locking piece. At last, new steel bar is mainly supported by the ribs instead of intercostal muscles in our procedure, which effectively relieve postoperative pain and reduce complications such as bar displacement caused by rupture of intercostal muscles. In our study, no rupture of intercostal muscles was found and all of the 1 cases of bar displacement after the correction were associated with fracture of ribs caused by violent collision during football game.

Due to the above reasons, it is easier and more convenient for surgeons to implant, fix and withdraw the bar. In our study, the time of bar implantation and the time of bar removal in the new procedure group were significantly shorter than those in the traditional procedure group. It was possible that the shortening of the operation time may be an important factor for less blood loss and rapid postoperative recovery. The hospital stay in the new procedure group was significantly shorter than that of the traditional procedure group. Nevertheless, the improvement of the new steel bar still followed the basic principles of bilateral incision, minimally invasive bar implantation, and sternal uplift, which might be an important reason for the non-statistical difference between the two groups in the length of incision, the cost, the postoperative Haller index and the postoperative surgical outcome.

Severe complication was not found in the new procedure group. Precious study illustrated that most postoperative wound infections should be treated conservatively by debridement after removing the fixation bar [16]. However, it was not necessary to remove the steel bar reported in our study. Bar displacement was often caused by rupture of intercostal muscles in traditional Nuss procedure, which may also the important reason for recurrence [15]. Nevertheless, no rupture of intercostal muscles was found in our study and that might because the bar was mainly supported by the ribs instead of intercostal muscles.

In our study, there were 4 recurrent PE and 7 PE patients with 2 steel bars in the new procedure group. A lot of experience was collected during operation for the PE patients above. Cardiac injury was the most severe complication for recurrent PE, as an intraoperative injury of the heart or a major blood vessel could lead to intraoperative or postoperative mortality [16]. As a result, the thoracoscopy was used and if necessary, an electrocautery hook was also introduced to dissect pleural adhesion in thoracic cavity followed by a 5-mm-diameter thoracoscope through each side of the chest. Moreover, our experiences showed that posterior sternal adhesion was usually severe and often difficult to be found in recurrent PE. Therefore, we routinely made a small incision under the xiphoid process to separate the adhesion between the right atrium and sternum under the guidance of thoracoscopy. In our study, no heart damage occurred, although right atrium was reported to be damaged during the separation process in several previous studies [17, 18]. If one bar was not enough to give a good cosmetic correction, another 1–2 bars were implanted through the same or additional incisions in previous studies [14].In the new procedure group of our study, 7 patients were implanted the second bar to correct the deformity, and no patient was treated by the third bar. Our experiences show those with 2 bars correction were often tall and thin, indicating that cases with lower body mass index (BMI) might tend to need 2 or more bars. The second bar was often implanted at the second or the third intercostal space with smaller size (than the first bar). We fixed steel bar with wires in the shape of an 8 and sutured the bar to the rib together with chest wall muscles so as to reduce the dislocation rate.

This study was limited by scale of the patients included. Further high-level clinical evidence is required to evaluate the long-term applicability and benefits of the use of introducer-bar complex.

## Conclusions

Our data show that the modified procedure for pectus excavatum may has shorter operation duration, postoperative hospitalization duration, plate removal surgery duration and less blood loss. Thus, the use of the modified procedure appears to be efficient and safe as it showed no increase in the incidence of complications.

## Supplementary Information

**Additional file 1.** A video of the Procedure of a modified Nuss operation using the introducer-bar complex for a 20-year-old PE patient.

**Additional file 2. Figure S1.** Introducers and bars according to the size.

**Additional file 3. Figure S3.** Appearance and chest scan of a 26-year-old patient with severe PE and scoliosis before and after modified Nuss procedure with introducer-bar complex.

## Data Availability

Data used for this study are available upon request.

## Abbreviations

PE: Pectus excavatum
NP: Nuss procedure
IQRs: Inter-quartile ranges
BMI: Body mass index
