# Supplementary figures and images for: Modified Nuss operation using introducer-bar complex for pectus excavatum in adults: a retrospective study
Source: J Cardiothorac Surg. 2021 Sep 22;16:267. doi: 10.1186/s13019-021-01624-6 (PMC8456631; doi:10.1186/s13019-021-01624-6)

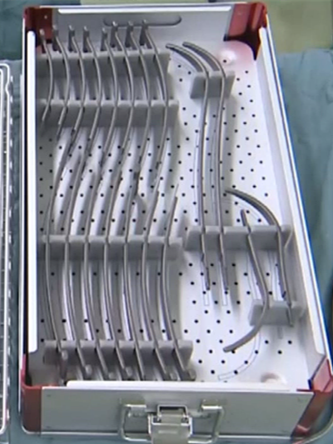

Supplement: Supplementary file 2 — Additional file 2. Figure S1. Introducers and bars according to the size. [file 13019_2021_1624_MOESM2_ESM.png]

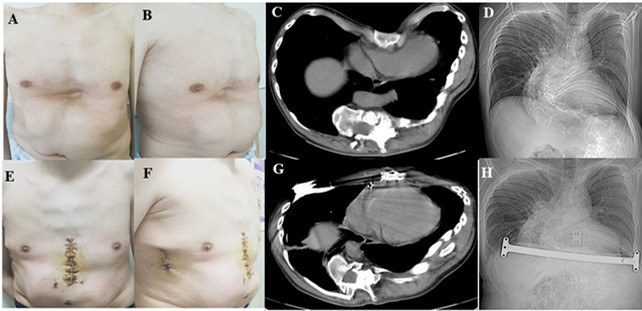

Supplement: Supplementary file 3 — Additional file 3. Figure S3. Appearance and chest scan of a 26-year-old patient with severe PE and scoliosis before and after modified Nuss procedure with introducer-bar complex. [file 13019_2021_1624_MOESM3_ESM.png]
